# One-Month Duration Compared with Twelve-Month Duration of Dual Antiplatelet Therapy in Elective Angioplasty for Coronary Artery Disease: Bleeding and Ischaemic Outcomes

**DOI:** 10.3390/jcm13154521

**Published:** 2024-08-02

**Authors:** Natasha Corballis, U. Bhalraam, Ioannis Merinopoulos, Tharusha Gunawardena, Vasiliki Tsampasian, Upul Wickramarachchi, Simon Eccleshall, Vassilios S. Vassiliou

**Affiliations:** 1 Centre of Metabolic Health, Norwich Medical School, University of East Anglia, Bob Champion Research and Education, Rosalind Franklin Road, Norwich NR4 7UQ, UKioannis.merinopoulos@nnuh.nhs.uk (I.M.);; 2 Department of Cardiology, Norfolk and Norwich University Hospital NHS Foundation Trust, Colney Lane, Norwich NR4 7LJ, UK; simon.eccleshall@nnuh.nhs.uk

**Keywords:** dual antiplatelet therapy, elective PCI, bleeding risk, DCB only PCI

## Abstract

**Background/Objectives**: The need to determine the safest duration of dual antiplatelet therapy duration after elective angioplasty to reduce bleeding events without an adverse effect on major adverse cardiovascular events (MACE) remains a challenge. **Methods**: In this investigator-initiated, single-centre cohort study, we identified all patients who underwent PCI for de novo coronary disease for stable angina between January 2015 and November 2019. We compared 1-month and 12-month durations of dual antiplatelet therapy (DAPT) to determine if there was any difference in the primary outcome of major bleeding. The secondary outcome was a patient-oriented composite endpoint of all-cause mortality; any myocardial infarction, stroke, or revascularisation; and the individual components of this composite endpoint. Data were analysed using Cox regression models and cumulative hazard plots. **Results**: A total of 1025 patients were analysed, of which 340 received 1 month of DAPT and 685 received 12 months of DAPT. There was no difference in major bleeding between the two groups (2.6% vs. 2.5% respectively). On univariable cox regression analysis, no characteristics were predictors of major bleeding. A proportion of 99.7% of patients in the 1-month DAPT arm were treated with a DCB strategy, whilst 93% in the 12-month DAPT group were treated with a DES. There was no difference between the two groups with regards to the composite patient-oriented MACE (11% vs. 12%, respectively) or any individual component of this. These results were unchanged after propensity score matched analysis. **Conclusions**: A 1-month duration of DAPT, for which 99.7% of patients were treated with a DCB strategy, appears safe and effective when compared with a 12-month duration of DAPT with no difference in major bleeding or MACE.

## 1. Introduction

There has been significant focus recently on determining a safe and short duration of dual antiplatelets for patients undergoing coronary intervention. This is partly driven by the increasingly co-morbid and aging population that presents with sequelae of ischaemic heart disease [1]. It is increasingly apparent that, whilst a long duration of dual antiplatelets therapy (DAPT) is associated with a reduction in ischaemic outcomes, this carries a risk of bleeding, morbidity and mortality [2]. Indeed, a 12-month duration of DAPT is associated with higher all-cause mortality and bleeding risk than a 6-month duration of DAPT [2]. In the acute coronary syndrome (ACS) cohort, a longer duration of DAPT is recommended by international guidelines [3]. However, in patients with stable coronary disease, assessment of an individual’s risk of bleeding is particularly encouraged to facilitate decision making regarding a percutaneous coronary intervention (PCI) strategy. The most comprehensive bleeding risk assessment tool is the PRECISE-DAPT scoring system, which is recommended by the ESC with a class IIb A recommendation for use [4].

The current guidelines recommend a 6-month duration of DAPT for all patients undergoing PCI for stable angina in sinus rhythm, although the P2Y12 inhibitor can be discontinued after 1–3 months if there is occurrence or high risk of a life threatening bleed [5]. Particularly in patients who have undergone stent implantation, the risk of early discontinuation of the P2Y12 inhibitor is an increased rate of stent thrombosis [4].

We sought to report bleeding outcomes and ischaemic safety outcomes for all patients undergoing elective PCI with a one-month duration of DAPT as compared with all patients receiving a 12-month course of DAPT.

## 2. Materials and Methods

All patients in a single centre undergoing PCI were prospectively entered into a clinical database. With the appropriate ethics (Northwest Haydock Research Ethics committee, UK 17/NW/0278) and Institutional Board approvals from Norfolk and Norwich University Hospital we retrospectively obtained clinical outcome measures from hospital episode statistics obtained from NHS Digital. The Confidentiality Advisory Group (CAG) waived the need for patient consent given the retrospective nature of our study. Our cohort was identified from 1 January 2015 until 15 November 2019 with all consecutive patients who underwent successful PCI for stable coronary disease included in this analysis. Successful PCI (defined as survival to the end of the procedure) and a definitive treatment strategy with either a 2nd generation DES or drug-coated balloon (DCB) was required for inclusion. The exclusion criteria were in-stent restenosis, atrial fibrillation and patients who received DAPT for >1 month and <12 month. Patients who opted out of hospital episode statistics (HES) follow-up were also excluded.

Clinical and angiographic data were obtained from our prospective database, supplemented by electronic hospital records when required. All angiograms were reviewed by an expert operator (NC, IM) to confirm accuracy of treatment strategy, and to classify bifurcation disease and lesion complexity. The vessel diameter was defined as the largest pre/post-dilatation balloon, drug-coated balloon (DCB) or drug eluting stent (DES) used, and the lesion length was based on the DCB or DES length.

Bleeding and ischaemic risk was calculated using the DAPT risk score, as this is a risk score designed for patients not on oral anticoagulation. The parameters used to calculate risk were age, diabetes, smoking within the last two years, previous MI or PCI, history of congestive heart failure or left ventricular ejection fraction <30%, hypertension, renal insufficiency, peripheral arterial disease, MI at presentation, stenting of a vein graft and stent diameter <3 mm [6].

The primary outcome was major bleeding at 12 months. All ICD-10 codes that could relate to bleeding events as defined by the Bleeding Academic Research Consortium (BARC) [7] that would fall within a bleeding event of type 3 or more were included. This is defined as the following:

- Type 3a: Overt bleeding plus haemoglobin drop of 3 to <5 g/dL (provided haemoglobin drop is related to bleed) or transfusion with overt bleeding
- Type 3b: Overt bleeding plus haemoglobin drop <5 g/dL (provided haemoglobin drop is related to bleed), cardiac tamponade, bleeding requiring surgical intervention for control, or bleeding requiring IV vasoactive agents
- Type 3c: Intracranial haemorrhage confirmed by autopsy, imaging or lumbar puncture, or intraocular bleed compressing vision
- Type 4: CABG related bleeding within 48 h
- Type 5a: Probable fatal bleeding
- Type 5b: Definite fatal bleeding (Overt or autopsy or imaging confirmation)

Our secondary endpoint was a patient-orientated composite outcome at 12 months, as recommended by the ARC-2 guidelines on device orientated outcomes [8], including the following:

- Any death
- Cerebrovascular event (CVE)
- Any myocardial infarction
- Any revascularisation

Twelve-month follow up was chosen as this is the point at which we would expect to see a difference in bleeding and ischaemic events as a direct consequence of duration of DAPT and because thereafter most patients on DAPT revert to single antiplatelet use.

All outcomes were obtained from the National Health Service Hospital Episode Statistics and Supplementary Table S1 outlines the ICD-10 diagnostic codes used to identify patient outcomes. The validated Hospital Frailty Risk Score, based on ICD-10 diagnostic codes, was used to calculate the patients’ frailty index [9]. An independent committee adjudicated the outcomes.

Statistical analysis was undertaken in R (version 4.2). Nominal variables are reported as counts (percentages) and compared using the Chi-square test. Variables that were not normally distributed, as assessed by the Kolmogorov and Shapiro tests, are reported as median (interquartile range). Univariable Cox regression analyses were undertaken to identify predictors of major bleeding and the composite major adverse cardiovascular event (MACE). Data are reported as hazard ratios (HRs) with 95% confidence intervals. A *p*-value of <0.05 was considered significant. Cumulative hazard plots were used to compare patient outcomes. Comparisons were performed by the log-rank test. A propensity score matching was subsequently undertaken to compare the 1-month and 12-month groups.

## 3. Results

A total of 1302 patients were initially identified for inclusion in analysis. However, 65 (4.9%) patients had opted out of HES data follow-up. After excluding patients with anticoagulant use or who had undergone DAPT for between 1 and 12 months, 1025 patients were included in the final analysis (340 receiving 1-month DAPT and 685 receiving 12-month DAPT) (Supplementary Figure S1, study consort diagram). The mean age was 68 (10) in the 1-month group and 67 (10) in the 12-month group. Females accounted for 23% of patients in the one-month group and 22% in the 12-month group. The groups were well balanced with regards to baseline characteristics, as shown in Table 1, with only COPD showing a difference between the two groups, with a higher number in the 12-month group.

The lesion/angiographic characteristics are outlined in Table 2. The groups were well balanced with regards to vessel-treated and multivessel PCI. Lesion complexity was higher in the one-month group, with significantly more calcification, tortuosity, diffuse disease and bifurcation lesions. The treatment strategy was markedly different between the two groups, as a DCB strategy was almost fully favoured in the one-month DAPT group (99.7%), whereas a DES strategy was the preferred in the 12-month group (93%). The treated vessel length and vessel diameter was significantly larger in the 12-month group.

The DAPT risk score was significantly higher in the 12-month DAPT group (19.7 v 12.4%, *p* = 0.04), suggesting an increased ischaemic risk in this group, while the number of those with a lower score (i.e., a higher likelihood of bleeding than ischaemia) was numerically higher but not statistically significant in the one-month group (87.6 v 80.3%, *p* = 0.05).

**Figure 1:**
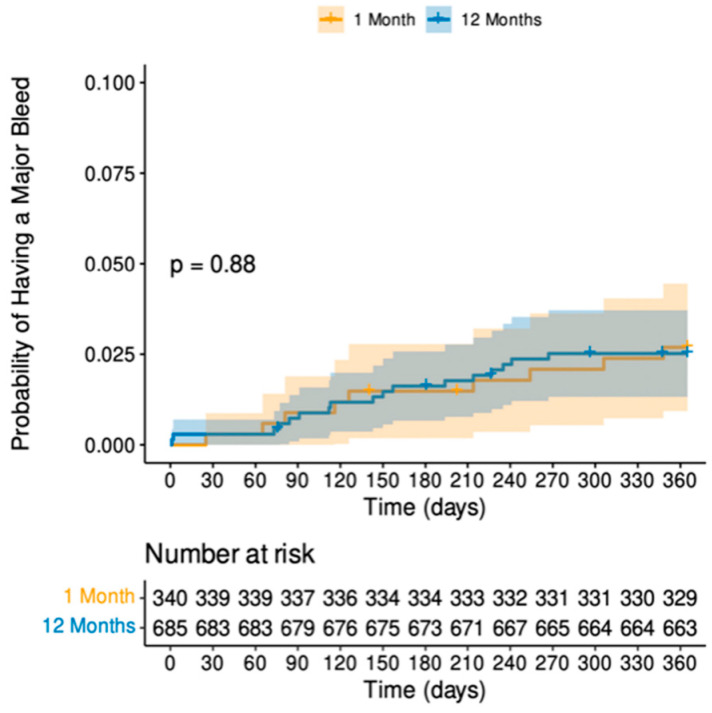
Cumulative hazard plot for major bleeding at one year. This shows no difference in the likelihood of major bleeding at one year, regardless of the one-month or twelve-month durations of DAPT.

Follow up was complete at 365 days for all patients still alive. There was no evidence in difference in bleeding events between the 1-month and 12-month durations of DAPT (*p* = 0.88), as shown by the cumulative hazard plot in Figure 1.

**Figure 2:**
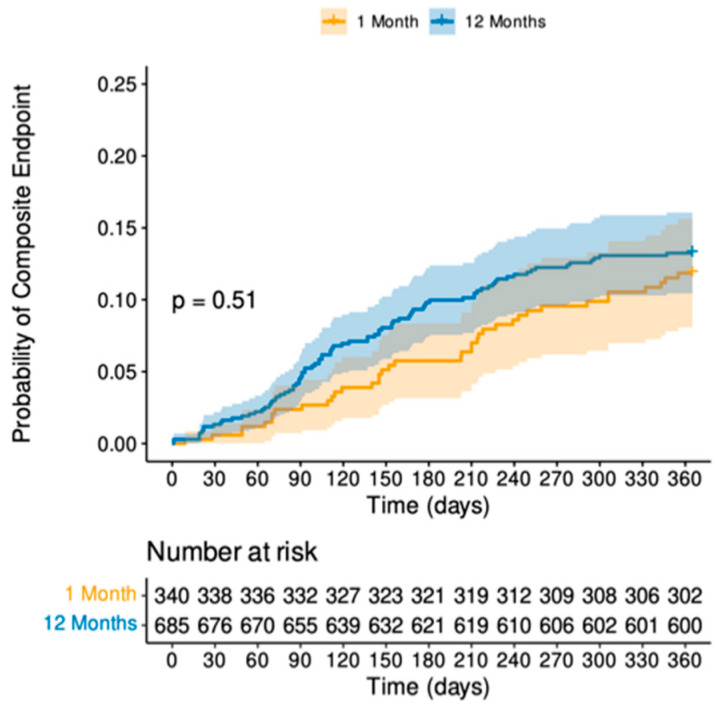
Cumulative hazard plot for secondary composite endpoint. This shows no difference between the one-month or twelve-month durations of DAPT with regards to the composite endpoint of any death, CVE, MI or any revascularisation.

Furthermore, no difference was identified between the two treatment strategies for the secondary composite patient-orientated safety endpoint at 1 year (Figure 2), with no difference in mortality (*p* = 0.99) (Supplementary Figure S1), any MI (0.22) (Supplementary Figure S2), CVA (*p* = 0.56) (Supplementary Figure S3), or any revascularisation (0.44) (Supplementary Figure S3) and as summarised in Table 3.

A subgroup analysis of those aged over 75 years of age showed no increased risk of major bleeding and high-risk DAPT score for ischaemia confirmed no difference between the two groups for a composite endpoint. Meanwhile, there was no increased risk of bleeding events in the patients who were at increased bleeding risk on the cumulative hazard plot.

Univariable cox regression analysis identified age >75 as the only independent predictor of bleeding events (Table 4) and a backward stepwise regression confirmed no variable to be an independent predictor of bleeding events. Univariable cox regression analysis for the secondary composite endpoint identified female sex, frailty, smoking status, heavy calcification and a graft lesion were predictors of mortality, MI, CVA and revascularisation (Table 5).

## 4. Discussion

This cohort study has shown no difference between bleeding or ischaemic events in 1025 patients, with 340 patients receiving one month and 685 patients receiving 12 month of DAPT. Surprisingly, this remained true even when analysing for high-risk patients, based on DAPT score, or for patients over the age of 75, although an age of over 75 was found to be an independent predictor of bleeding event rates on univariate analysis. This is an expansion of a previous cohort analysis [10] and reflects the patients with stable angina that have been previously reported [11]. We have demonstrated that this is overall a low bleeding risk population, partially by way of excluding anticoagulant use in the analysis. However, and importantly, we have shown no difference in ischaemic risk, regardless of whether DAPT was given for a 1-month or 12-month duration. This may be driven by the almost exclusive use of DCB in the 1-month DAPT duration group, suggesting that the lack of a stent implant may be of benefit in reducing ischaemic events with a 1-month DAPT duration.

Bleeding is increasingly being recognised as a significant cause of morbidity and mortality in patients undergoing PCI. Current guidelines, including the recent ACC/AHA 2023 guidelines [12], still routinely recommend a minimum of 6 months of DAPT [5]; however, though there has been a recognition of more personalised DAPT duration, based on the individual’s bleeding risk, with guidelines recognising that a 1–3-month duration of DAPT is acceptable in patients at high risk of bleeding. The concerns surrounding stent thrombosis risk by reducing DAPT from 12 months to 6 months were allayed by a meta-analysis showing that, whilst this shorter duration of DAPT was associated with an increased risk of MI and stent thrombosis, there was no corresponding increase in cardiovascular mortality and that, indeed, all-cause mortality was lower in the 6-month group [13]. This has led to a paradigm shift in offsetting bleeding risk with ischaemic risk by optimising the duration of DAPT after PCI.

Bleeding events contribute significantly to comorbidity and mortality in patients undergoing PCI and a number of RCTs may underestimate true rates of bleeding events due to stringent inclusion criteria that exclude patients who would be at a higher risk of bleeding [14]. A recent analysis of the NORSTENT data showed a twelve-month cumulative BARC major bleeding rate of 2.3% [15], which is higher than contemporaneous studies but still excludes patients with a contra-indication to DAPT or who are on any anticoagulants. A large prospective registry analysis of 13258 patients showed that, in the patients undergoing PCI for stable coronary disease, major bleeding events within 30 days of PCI was 3% and a 5-year cumulative event rate was at 15.2% [16]. This analysis included patients with a high risk of bleeding and is likely a more accurate reflection of the likelihood of bleeding rates in current practice. The major bleeding rates in our study are comparably lower and this may be due to the fact that our cohort does not represent a population with a high risk of bleeding—we excluded AF with concomitant use of anticoagulants, the mean age of patients included was 67.5 and the number of patients with frailty were low.

Determining the safety of a one-month duration of DAPT has been an increasing research focus. The ZEUS [17] (high bleeding risk population, MACE at twelve months of 17.5%), SENIOR [18] (>75 years of age, MACE at twelve months of 12%) and LEADERS-FREE [19] trials (high bleeding risk, MACE at twelve months of 9.4%) have all established that DES are superior to bare metal stents (BMS) as part of a one-month duration of DAPT. Subsequent studies have included the DAPT trial [20] (one-month versus six-month DAPT in ACS and stable coronary disease), which reported a MACE of 5.9% at one year in the one-month DAPT arm; the Onyx-One trial [21] (one month duration of DAPT in ACS and stable coronary disease), which reported a MACE rate of 16.9% and the STOP-DAPT-2 trial, which compared one-month and twelve-month durations of DAPT in ACS and stable coronary disease and reported a MACE rate of 2.36%. This MACE rate is significantly lower than any of the previously mentioned trials that had a one-month duration of DAPT and a possible explanation for this is that intra-coronary imaging was used in all cases in the STOP-DAPT 2 trial. There has increasingly been a move to investigate a short duration of DAPT followed by P2Y12 inhibitor monotherapy, in both chronic coronary syndromes and ACS patients [22,23], with a number of trials currently recruiting in STEMI/ACS patients (BULK-STEMI, COMPARE STEMI ONE, ULTIMATE DAPT, MATE, TARGET FIRST) [24]. The results of these trials may alter our approach to the prolonged use of aspirin post PCI but, as these studies are associated with a more potent P2Y12 inhibitor, this may limit uptake in patients with chronic coronary syndromes. However, it is likely that PCI results in an inflammatory response, which aspirin may have a role in treating [25,26].

The selection of the P2Y12 inhibitor has also been an important research topic. The guidelines routinely recommend clopidogrel [4]. The use of cangrelor for complex elective patients has recently been studied for P2Y12-naïve patients, and this has shown that clopidogrel, when administered after cangrelor, exposes some patients to a period of inadequate platelet inhibition [27] This adds weight to the notion of personalizing antiplatelet regimens based not only on a patient’s individual risk factors/bleeding risk but also on the complexity of the PCI strategy.

The majority of patients in this study who were receiving a one-month duration of DAPT were treated with a DCB. This reflects the practice of a centre with significant experience in DCB-only angioplasty [28,29,30]. Whilst current guidelines recommend DCB in in-stent restenosis only [31], there is increasing evidence supporting their use in de novo coronary disease [29,30,31,32,33], with RCT evidence showing non-inferiority in small vessel disease [34]. A previous analysis of safety for one-month durations of DAPT in our centre showed a 0% occurrence of MACE at 6 months [10]. The DEBUT trial [35] (DCB versus BMS in high-risk-of-bleeding patients) reported a MACE rate of 0%. The only DES study to have a comparably low MACE rate involved intra-coronary imaging to optimise stent implantation in all cases [36]. Direct comparisons cannot be drawn between these studies due to the heterogeneity of the study design, the inclusion criteria and the patient demographics; however, it is clear that DCBs are a safe alternative to DES implantation in a patient who is at high risk of bleeding. Whilst our MACE rates are not as low as previously reported, this may be due to our adoption of a patient-orientated composite endpoint. Whilst this is more applicable and relevant to our patient population, its definition has a broader reach that encompasses all-cause mortality and any revascularisation (both planned and unplanned), which may explain the higher MACE rate in this cohort.

If part of the concern of shortening DAPT in our elective PCI patients is due to concerns of stent thrombosis, DCB can be an attractive proposition. There is already evidence supporting the use of DCBs in de novo lesions in small vessel disease [34] and there is increasing evidence supporting their use in STEMIs, ACS and coronary bifurcations not limited to small vessels [32]. Our results confirm that a DCB approach with one-month duration of DAPT is not associated with any increased ischaemic risk. We believe that we did not see any reduction in bleeding risk in the one-month duration DAPT cohort as this group was made up of a population with low bleeding risk. Most importantly, perhaps, is the role of DCBs in personalised medicine. In an elective patient population, where we can determine bleeding risk prior to the procedure, the use of an intended DCB strategy can minimise duration of DAPT to one month without the inference of a concern for stent thrombosis.

### Limitations

As a retrospective registry analysis, the inherent risk of selection bias and confounding errors are ever present. However, this is countered by a reflection of real-world data analysis, which provides a truer representation of our patient population in everyday practice. Bias in this patient population is reduced by including all consecutive patients in our centre, which reflects a catchment area of more than 1.5 million people. Although this study is a retrospective analysis, all patients are entered prospectively into the clinical database and the groups were largely well balanced in terms of patient and angiographic characteristics.

Whilst the majority of studies report major bleeding to be BARC 3 or more, a type 2 bleeding event is still an actional haemorrhage requiring diagnostic studies and/or hospital admission. Based on our access to HES data, we could not define all of these events and so these will not have been captured in this analysis, an aspect which should be recognized as a limitation.

We express an intended treatment strategy, and the subsequent change in DAPT regimen cannot be reflected in this analysis given the retrospective nature.

The use of intravascular imaging was low in this cohort. This reflects UK trends during the timeframe of the analysis [37] but may limit the generalizability of results to contemporary practice.

The use of the DAPT risk score was initially introduced to determine ischaemic v. bleeding risk at beyond 12 months when considering a prolonged duration of DAPT, although it has subsequently been validated by meta-analysis to show efficacy in shorter duration DAPT studies and prediction of event rates [6].

As patients have the opportunity to opt out of HES data follow up, 4.9% of patients initially identified for inclusion could not be analysed due to lack of availability of the HES data.

Finally, the majority of patients receiving a one-month duration of DAPT were treated with a DCB, suggesting that operators may have a bias towards a DCB strategy in patients for which there are concerns regarding bleeding risk.

## 5. Conclusions

This study demonstrates that, whilst there was no difference in major bleeding between a one-month and twelve-month duration of DAPT, this was, reassuringly, not associated with an increased rate of patient-orientated major adverse cardiovascular events. The patients treated with a one-month duration of DAPT were predominantly treated with a DCB strategy, suggesting that this is a safe and effective method of reducing DAPT duration with no impact on ischaemic events. With age identified as an independent predictor of bleeding event rates, our work further suggests that a DCB strategy is an important consideration for our aging population for reducing adverse bleeding events and is not associated with an increased risk of ischaemia in this complex sub-group of patients. The clinical equipoise observed in this retrospective analysis highlights the need for a randomized controlled trial.

## Figures and Tables

**Table 1:** Baseline characteristics.

Patient Characteristics	One-Month DAPT (*n* = 340)	Twelve-Month DAPT (*n* = 685)	*p* Value
Female sex	78 (22.9)	154 (22.5)	0.9
Age (mean, SD)	68 (10)	67 (10)	0.087
Frailty score (median, IQR)	0.0 (0.0–0.7)	0.0 (0.0–0.6)	0.35
Frailty - Low - Intermediate - High	323 (99.7) 1 (0.3) 0 (0)	642 (99.4) 4 (0.6) 0 (0)	0.4
Hypertension	186 (54.7)	392 (57.2)	0.4
Dyslipidaemia	115 (33.8)	231 (33.7)	0.9
Previous CVE	23 (6.8)	32 (4.7)	0.1
Peripheral vascular disease	14 (4.1)	29 (4.2)	0.7
Previous MI	45 (13.2)	116 (16.9)	0.1
Previous PCI	51 (15.0)	86 (12.6)	0.3
Previous CABG	29 (8.5)	49 (7.2)	0.4
COPD	11 (3.2)	42 (6.1)	**0.05**
Family history of coronary disease	91 (26.8)	178 (26.0)	0.8
Diabetic	78 (22.9)	155 (22.6)	0.9
Current/ex-smoker	212 (62.7)	457 (67.3)	0.1
Dual antiplatelet use	340 (100)	685 (100)	>0.99
DAPT Risk Score			
<2	296 (87.6)	545 (80.3)	0.05
≥2	42 (12.4)	134 (19.7)	**0.04**
DAPT used			
Aspirin and clopidogrel	329 (96.7)	630 (92)	
Aspirin and ticagrelor	7 (2.1)	50 (7.3)	
Aspirin and prasugrel	4 (1.2)	5 (0.7)	

SD = standard deviation, IQR = interquartile range, CVE = cerebrovascular event, MI = myocardial infarction, PCI = percutaneous coronary intervention, CABG = coronary artery bypass grafting, COPD = chronic obstructive pulmonary disease, DAPT = dual antiplatelet therapy. A statistically significant *p*-value is highlighted in bold.

**Table 2:** Angiographic characteristics of treated lesions.

Lesion Characteristics	Duration of DAPT		*p*-Value
	1 Month (*n* = 340)	12 Months (*n* = 685)	
Vessel treated, *n* (%)			0.054 ^1^
LMS	12 (3.5)	25 (3.6)	
LAD	192 (56)	375 (55)	
Cx	64 (19)	96 (14)	
RCA	69 (20)	170 (25)	
Graft	3 (0.9)	19 (2.8)	
Multivessel PCI, *n* (%)	32 (9.4)	88 (13)	0.11 ^1^
Heavy calcification, *n* (%)	104 (31)	153 (22)	**0.004** ^1^
Severe tortuosity, *n* (%)	79 (23)	86 (13)	**<0.001** ^1^
Diffuse disease	118 (35)	139 (20)	**<0.001** ^1^
DCB/DES use, *n* (%)			**<0.001** ^1^
DCB	339 (99.7)	45 (6.6)	
DES	1 (0.3)	640 (93.4)	
Vessel diameter, median (IQR)	3.00 (2.75–3.50)	3.50 (3.00–4.00)	**<0.001** ^1^
Vessel length, median (IQR)	20 (20–30)	24 (18–38)	**0.019** ^1^
Bifurcation lesion	104 (31)	143 (21)	**<0.001** ^1^
Intravascular imaging	8 (2.4)	61 (8.9)	**0.04** ^1^

^1^ Pearson’s chi-squared test; LMS = left main stem, LAD = left anterior descending, Cx = circumflex, RCA = right coronary artery. A statistically significant *p*-value is highlighted in bold.

**Table 3:** Summary of primary and secondary endpoints.

Outcomes, *n* (%)	Duration of DAPT 1 Month, *n* = 340	12 Months, *n* = 685	*p*-Value
Major bleeding	9 (2.6)	17 (2.5)	0.87 ^1^
All-cause mortality	3 (0.9)	6 (0.9)	>0.99 ^2^
Cardiovascular mortality	1 (0.3)	1 (0.1)	0.55 ^2^
ACS	8 (2.4)	9 (1.3)	0.22 ^1^
Cerebrovascular event	2 (0.6)	2 (0.3)	0.60 ^2^
Any revascularisation	31 (9.1)	72 (11)	0.48 ^1^
Composite of mortality, ACS, CVE and revascularisation	38 (11)	85 (12)	0.57 ^1^

^1^ Pearson’s chi-squared test, ^2^ Fisher’s exact test. ACS = acute coronary syndrome, CVE = cerebrovascular event.

**Table 4:** Univariable cox regression analysis for major bleeding.

Characteristic	Hazard Ratio (95% CI)	*p* Value
Age	1.02 (0.99–1.06)	0.21
Sex [Female]	0.42 (0.13–1.37)	0.15
DCB/DES use [DES]	1.07 (0.51–2.24)	0.86
Frailty score	0.77 (0.45–1.32)	0.34
Dyslipidaemia	0.84 (0.38–1.86)	0.67
Hypertension	0.61 (0.29–1.27)	0.19
Cerebrovascular event	0.57 (0.08–4.21)	0.58
Myocardial infarction	1.58 (0.67–3.69)	0.29
Coronary artery bypass grafting	1.92 (0.67–5.50)	0.23
Heart failure	1.56 (0.21–11.4)	0.66
Chronic obstructive pulmonary disease	0.69 (0.09–5.11)	0.72
Diabetes mellitus	1.06 (0.45–2.48)	0.89
Previous/current smoker	0.89 (0.42–1.88)	0.76
Creatinine	1.00 (0.99–1.02)	0.55
Multivessel disease	2.03 (0.83–4.98)	0.12
Bifurcation lesion	0.71 (0.27–1.87)	0.49
Heavy calcification	0.74 (0.30–1.81)	0.50
Diffuse disease	0.74 (0.30–1.82)	0.51
Severe tortuosity	0.60 (0.18–2.00)	0.41
Vessel diameter	0.63 (0.34–1.17)	0.14
Lesion length	1.00 (0.97–1.02)	0.78

**Table 5:** Univariable cox regression analysis for composite secondary endpoint.

Characteristic	Hazard Ratio (95% CI)	*p* Value
Age	1.01 (1.00–1.03)	0.16
Sex [Female]	0.61 (0.40–0.92)	0.01
DCB/DES use [DES]	0.84 (0.62–1.13)	0.24
Frailty score	1.08 (0.94–1.25)	0.27
Dyslipidaemia	1.12 (0.82–1.52)	0.48
Hypertension	1.01 (0.75–1.37)	0.93
Cerebrovascular event	1.48 (0.86–2.55)	0.16
Myocardial infarction	1.14 (0.78–1.67)	0.49
Coronary artery bypass grafting	1.02 (0.59–1.76)	0.95
Heart failure	1.33 (0.55–3.24)	0.53
Chronic obstructive pulmonary disease	0.67 (0.30–1.51)	0.33
Diabetes mellitus	1.36 (0.98–1.86)	0.06
Previous/current smoker	0.73 (0.54–0.99)	**0.04**
Creatinine	1.00 (1.00–1.01)	0.12
Multivessel disease	1.04 (0.66–1.65)	0.85
Bifurcation lesion	1.19 (0.85–1.65)	0.85
Heavy calcification	1.76 (1.29–2.39)	<0.001
Diffuse disease	1.14 (0.82–1.59)	0.42
Severe tortuosity	1.06 (0.71–1.58)	0.77
Vessel diameter	1.00 (0.78–1.28)	>0.99
Lesion length	1.00 (0.99–1.01)	0.66

Propensity score matching revealed the same results for all analyses and is included in the Supplementary Materials (Supplementary Figures S4–S7). A statistically significant *p*-value is highlighted in bold.

## Data Availability

The original contributions presented in the study are included in the article/Supplementary Materials, further inquiries can be directed to the corresponding author/s.

## Supplementary Materials

The following supporting information can be downloaded at: https://www.mdpi.com/article/10.3390/jcm13154521/s1, Table S1: ICD-10 codes used to determine clinical outcomes; Figure S1: Study consort diagram; Figure S2: Cumulative hazard plot for all-cause mortality and ACS; Figure S3: Cumulative hazard plot for CVA and revascularisation; Figure S4: Distribution of propensity scores for DES and DCB units; Figures S5–S7: Propensity matched cumulative hazard plots.
