# Supplementary material for: One-Month Duration Compared with Twelve-Month Duration of Dual Antiplatelet Therapy in Elective Angioplasty for Coronary Artery Disease: Bleeding and Ischaemic Outcomes
Source: J Clin Med. 2024 Aug 2;13(15):4521. doi: 10.3390/jcm13154521 (PMC11312761; doi:10.3390/jcm13154521)
Supplement: Supplementary file 1 [file jcm-13-04521-s001.zip › jcm-3122787-supplementary.pdf]

Supplementary table 1: ICD-10 codes used to determine clinical outcomes

**Major bleeding**	
Subarachnoid haemorrhage	I60*
Intracerebral haemorrhage	I61*
Non-traumatic intracranial bleeding	I62*
Gastrointestinal bleeding	K920, K921, K922
Additional GI bleeding	K25*, K26*, K27*, K28*, K29*, I85*
Haematuria	N02*, R31X
Bleeding complicating PCI	I97410, I97610
Bleeding complicating CABG	I97411, I97611, I976*, I974*
Haemorrhage not elsewhere specified	R58X
**ACS**	
STEMI	I210*, I211*, I212*, I213
NSTEMI	I214, I219, I200 (UA)
Re-infarction	I22*
**Revascularisation**	
PCI	K49*, K50*, K75*
CABG	K40*, K45*, K46*
**CVA**	
Acute ischaemic stroke	I63*
Haemorrhagic stroke	I61*
TIA/ stroke syndromes	I64X, G453, G459, G46*

Supplementary Figure 1: Study consort diagram

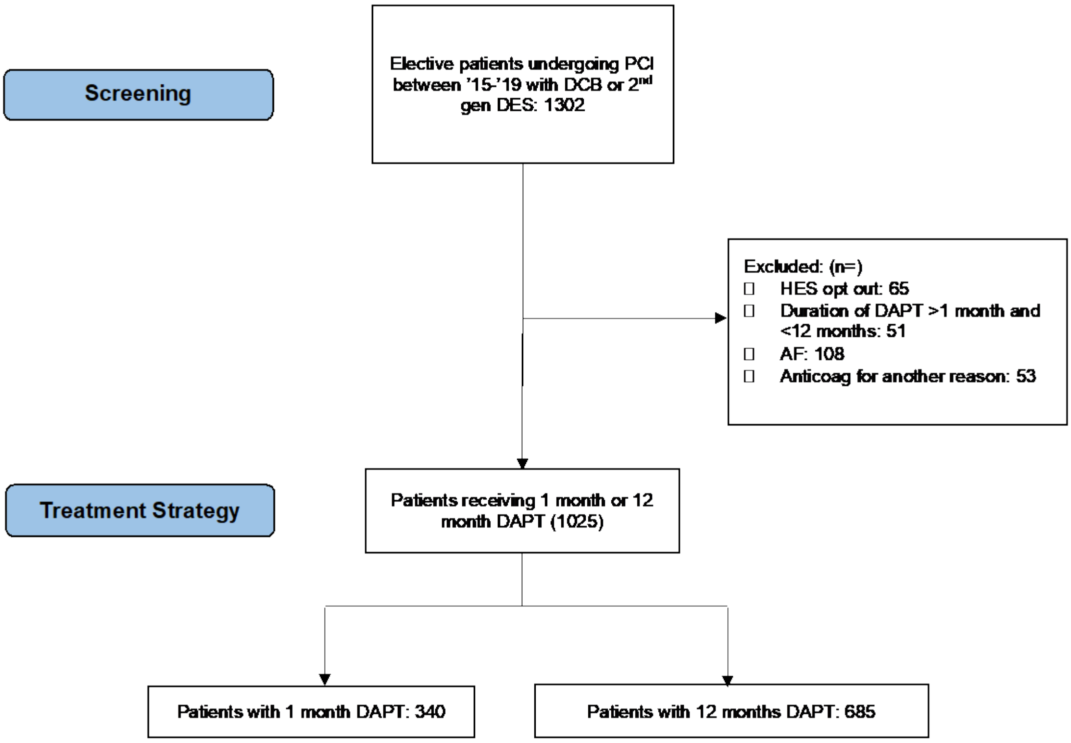

Supplementary Figure 2: Cumulative hazard plot for all-cause mortality and ACS

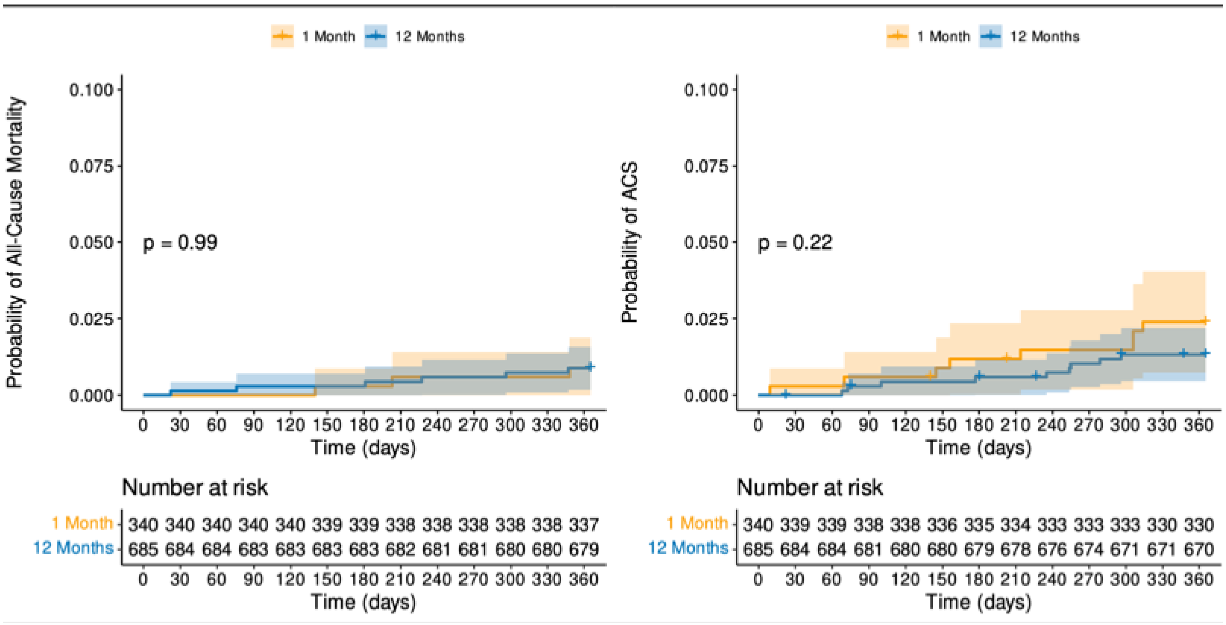

Supplementary Figure 3: Cumulative hazard plot for CVA and revascularisation

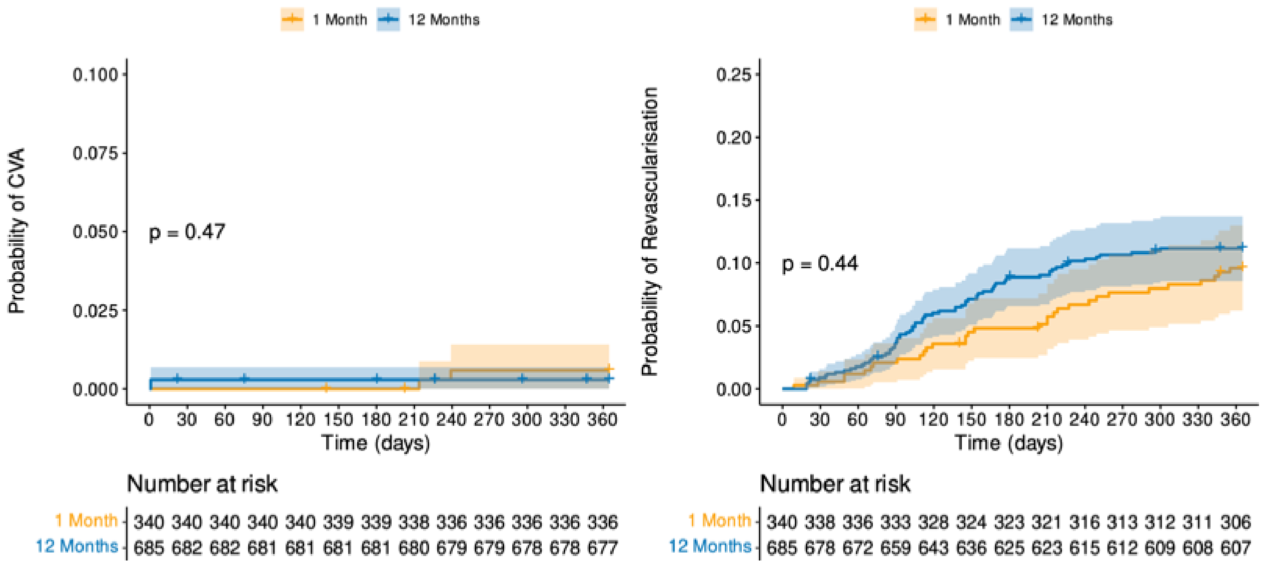

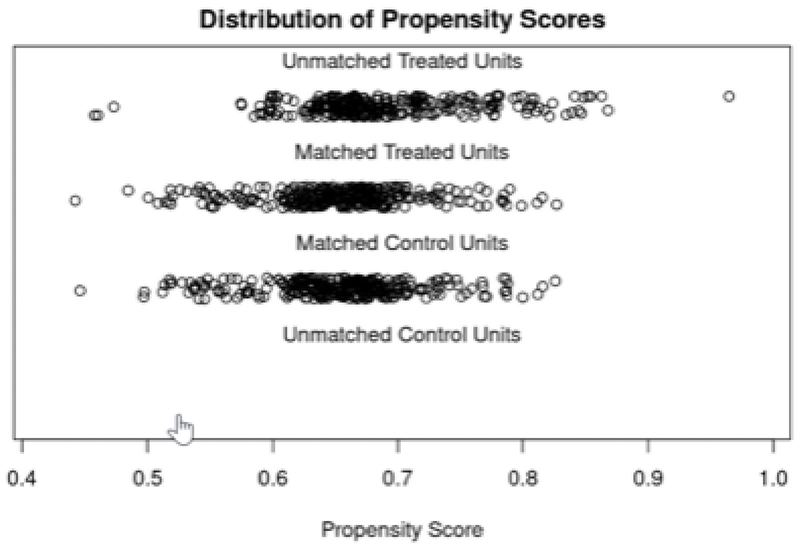

Supplementary Figure 4: Distribution of propensity scores for DES and DCB units

This figure illustrates the distribution of propensity scores for DES and DCB units before and after matching. The plot shows the distribution of scores for unmatched DES units, matched DES units, matched DCB units and unmatched DCB units.

Supplementary Figures 5-7: Propensity-matched cumulative hazard plots

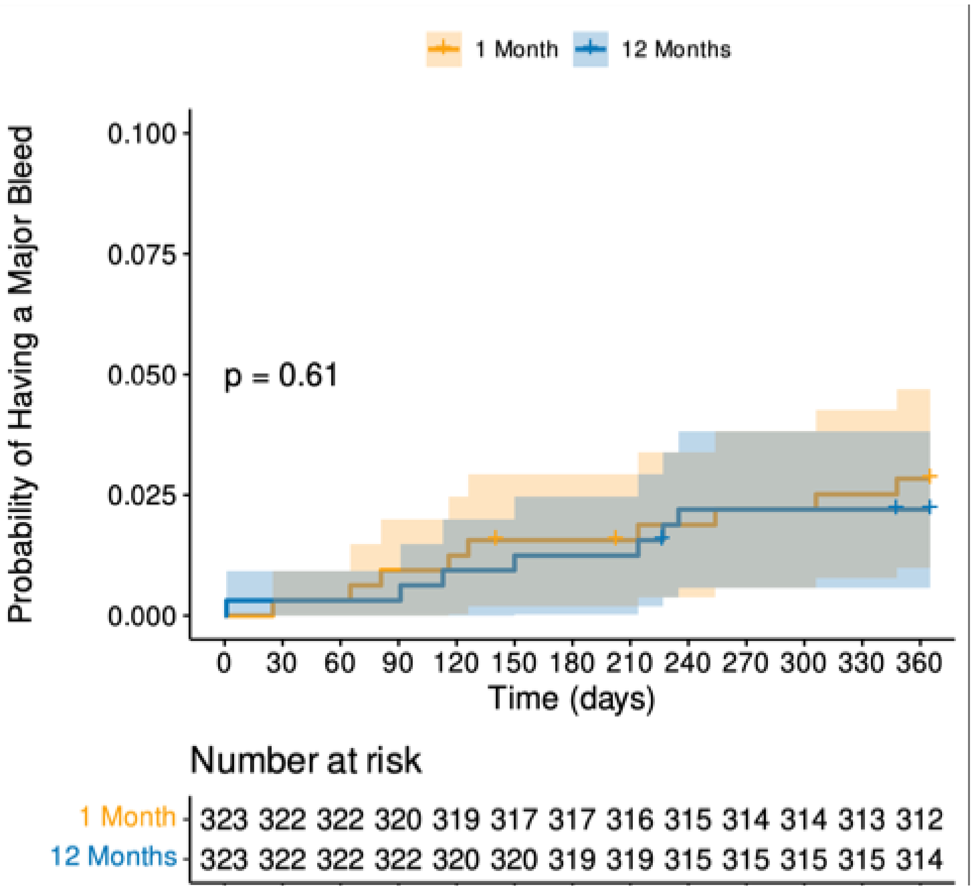
1 Month
12 Months
Probability of Having a Major Bleed
0.100
0.075
0.050
0.025
0.000
p = 0.61
0 30 60 90 120 150 180 210 240 270 300 330 360
Time (days)
Number at risk
1 Month 323 322 322 320 319 317 317 316 315 314 314 313 312
12 Months 323 322 322 322 320 320 319 319 315 315 315 315 314

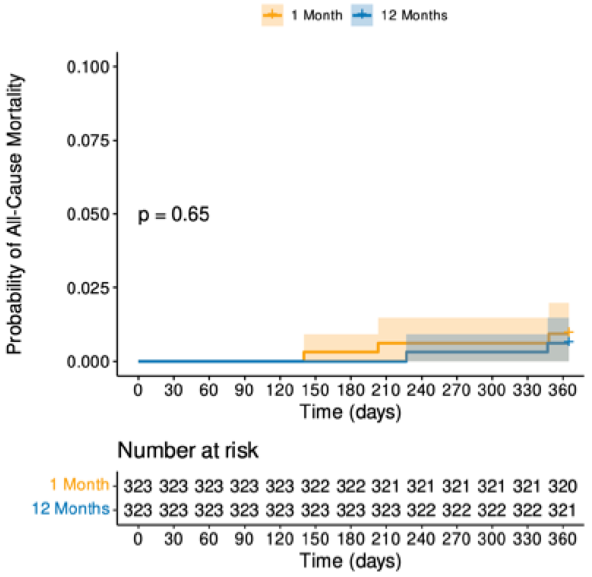
1 Month
12 Months
Probability of All-Cause Mortality
0.100
0.075
0.050
0.025
0.000
p = 0.65
0 30 60 90 120 150 180 210 240 270 300 330 360
Time (days)
Number at risk
1 Month 323 323 323 323 323 322 322 321 321 321 321 321 320
12 Months 323 323 323 323 323 323 323 323 322 322 322 322 321
0 30 60 90 120 150 180 210 240 270 300 330 360
Time (days)

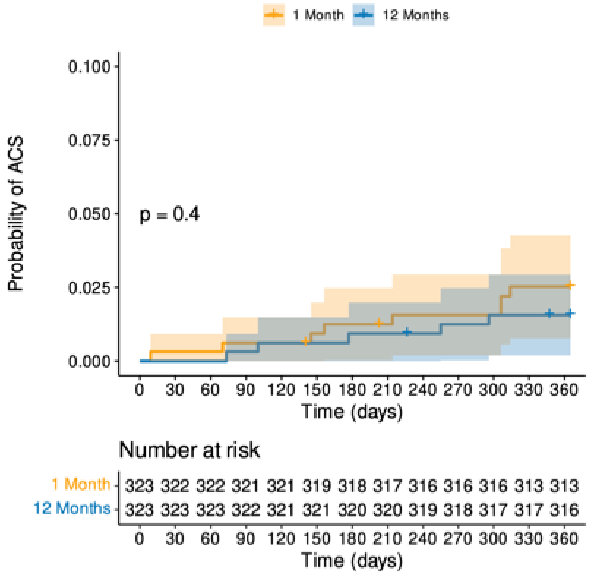
1 Month
12 Months
Probability of ACS
0.100
0.075
0.050
0.025
0.000
p = 0.4
0 30 60 90 120 150 180 210 240 270 300 330 360
Time (days)
Number at risk
1 Month 323 322 322 321 321 319 318 317 316 316 316 313 313
12 Months 323 323 323 322 321 321 320 320 319 318 317 317 316
0 30 60 90 120 150 180 210 240 270 300 330 360
Time (days)

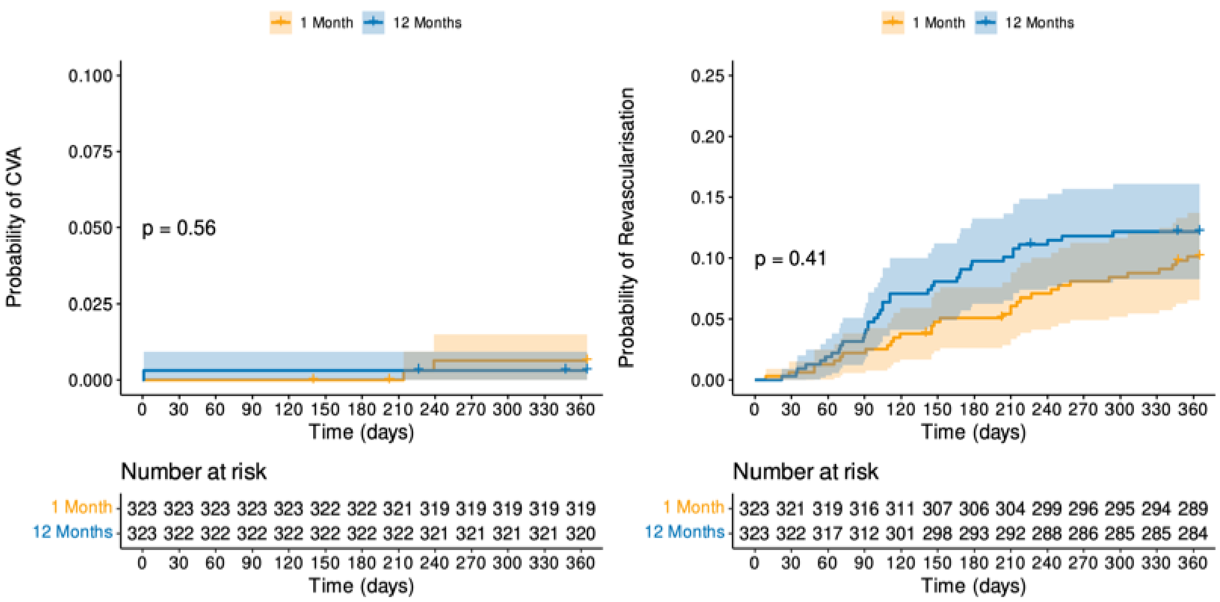
1 Month
12 Months
Probability of CVA
p = 0.56
0.100
0.075
0.050
0.025
0.000
0 30 60 90 120 150 180 210 240 270 300 330 360
Time (days)
Number at risk
1 Month 323 323 323 323 323 322 322 321 319 319 319 319 319
12 Months 323 322 322 322 322 322 322 322 321 321 321 321 320
0 30 60 90 120 150 180 210 240 270 300 330 360
Time (days)
1 Month
12 Months
Probability of Revascularisation
p = 0.41
0.25
0.20
0.15
0.10
0.05
0.00
0 30 60 90 120 150 180 210 240 270 300 330 360
Time (days)
Number at risk
1 Month 323 321 319 316 311 307 306 304 299 296 295 294 289
12 Months 323 322 317 312 301 298 293 292 288 286 285 285 284
0 30 60 90 120 150 180 210 240 270 300 330 360
Time (days)
